# The role of the BK channel in ethanol response behaviors: evidence from model organism and human studies

**DOI:** 10.3389/fphys.2014.00346

**Published:** 2014-09-09

**Authors:** Jill C. Bettinger, Andrew G. Davies

**Affiliations:** Department of Pharmacology and Toxicology, Virginia Commonwealth UniversityRichmond, VA, USA

**Keywords:** BK, *slo*, KCNMA1, *slo-1*, potassium channel, ethanol, genetics

## Abstract

Alcohol abuse is a significant public health problem. Understanding the molecular effects of ethanol is important for the identification of at risk individuals, as well as the development of novel pharmacotherapies. The large conductance calcium sensitive potassium (BK) channel has emerged as an important player in the behavioral response to ethanol in genetic studies in several model organisms and in humans. The BK channel, *slo-1*, was identified in a forward genetics screen as a major ethanol target in *C. elegans* for the effects of ethanol on locomotion and egg-laying behaviors. Regulation of the expression of the BK channel, *slo*, in *Drosophila* underlies the development of rapid tolerance to ethanol and benzyl alcohol sedation. Rodent expression studies of the BK-encoding *KCNMA1* gene have identified regulation of mRNA levels in response to ethanol exposure, and knock out studies in mice have demonstrated that the β subunits of the BK channel, β1 and β4, can modulate ethanol sensitivity of the channel in electrophysiological preparations, and can influence drinking behavior. In human genetics studies, both *KCNMA1* and the genes encoding β subunits of the BK channel have been associated with alcohol dependence. This review describes the genetic data for a role for BK channels in mediating behavioral responses to ethanol across these species.

## Introduction

Alcohol abuse is a significant worldwide socioeconomic problem, for which there are very few treatments. Despite the severity and prevalence of the disease, the molecular mechanisms by which alcohol exerts the effects that are relevant to drinking behavior remain incompletely understood. One major difficulty has been identifying behaviorally relevant ethanol targets. Ethanol interacts with many molecules *in vitro*, but translating information into an understanding of the behavioral relevance of the interactions requires different experimental approaches.

It is clear that there are physiological differences between individuals that influence their susceptibility to developing an alcohol use disorder, and that these differences are genetically influenced (Rodriguez et al., 1993; Schuckit and Smith, 1996; Prescott and Kendler, 1999). This indicates that there are differences in the human population in the neurobiological response to alcohol, and that these differences underlie at least some of the risk to abuse the drug.

One approach to identifying the mechanisms by which ethanol mediates its effects on behavior is to use genetic analysis, which allows for *in vivo* examination of the effect of ethanol on the function of a gene in mediating a behavioral phenotype. There are several types of genetic analysis, which can be divided into two major categories, forward and reverse genetics. In forward genetic analysis, the functions of all genes in a genome are examined using large-scale mutant screens. One important aspect of this approach is that there are no assumptions made about the relevance of any particular gene, and genes emerge from this analysis only if they have detectable effects on the phenotype being studied. Reverse genetics involves examining the function of particular targets *in vivo* by manipulating the expression or function of the gene. Reverse genetics allows for exquisite dissection of the function of particular genes in a phenotype of interest.

Model organisms, such as *Caenorhabditis elegans* and *Drosophila melanogaster*, provide powerful genetic tools, short generation times and relatively simple nervous systems with which to approach the problem of identifying the targets of a neuroactive drug and the physiological responses associated with the development of tolerance to such a drug (Schafer, 2004; Giacomotto and Ségalat, 2010; Devineni and Heberlein, 2013). Genetic analysis in rodent models is more laborious, but rodents allow for the genetic analysis of much more elaborate nervous systems, as well as very complex behaviors, such as voluntary ethanol drinking.

Using forward and reverse genetics approaches, many different laboratories across several different model organisms and humans have identified the large conductance voltage and calcium-sensitive potassium (BK) channel as being important in modulating the behavioral effects of ethanol. The purpose of this review is to describe genetic evidence supporting a major role for BK as a central mediator of ethanol's effects on behavior.

### *C. elegans*: identification of BK as the major mediator of the depressive behavioral effects of ethanol

One of the most powerful approaches for the identification of novel genes that are important for a phenotype is a forward genetic screen (Jorgensen and Mango, 2002). Wild-type animals are treated with a mutagen to induce random mutations in the germline and subsequent progeny bearing the induced mutations are screened for a phenotype of interest, such as reduced sensitivity to the effects of a drug. Such an approach, in theory, can assess the *in vivo* impact of a mutation in every gene on the phenotype of interest and it does so with minimal bias and no *a priori* assumptions about which gene is important. We used this approach with *C. elegans* to identify potential direct targets of ethanol using a behavioral response as the means of selection.

In a screen for mutant animals that were less sensitive to the depressive effects of ethanol on the speed of locomotion, we identified multiple independent mutations in the *slo-1* gene (Davies et al., 2003), the *C. elegans* homolog of the mammalian *KCNMA1* gene (Wang et al., 2001), which encodes the α subunit of the BK channel. The *slo-1* mutants had the largest level of ethanol resistance for any mutant found, suggesting that with loss of *slo-1*, a significant effect of ethanol had been blocked. In this genetic screen of randomly induced mutations, the mutations in *slo-1* were identified without bias from the findings that the BK channel was already known to be a target of ethanol based on numerous *in vitro* assays (Dopico et al., 1996, 1998, 1999; Jakab et al., 1997; Chu et al., 1998; Walters et al., 2000; Gruss et al., 2001). In *C. elegans*, the BK channel is expressed in neurons and in muscle (Wang et al., 2001). We confirmed a requirement for BK channels in the nervous system for ethanol to produce its effects by expressing wild-type SLO-1 only in neurons in an otherwise *slo-1* null mutant background and restoring wild-type sensitivity to the effects of ethanol on locomotion speed (Davies et al., 2003). Electrophysiology was performed on intact *C. elegans* neurons, which showed that ethanol increased BK channel currents but failed to do so in *slo-1* null mutant neurons (Davies et al., 2003). Two gain-of-function *slo-1* mutants displayed phenotypes that were characteristic of ethanol intoxication, providing further confirmation that hyperactivation of BK channels can produce intoxicated behaviors (Davies et al., 2003).

From the same genetic screen, mutations that affected members of the dystrophin-associated protein complex (DAPC) were also identified as being resistant to the effects of ethanol (Davies et al., 2003). These mutants did not have the same level of ethanol resistance as *slo-1* mutants, but they did share certain basal (observed in the absence of ethanol) mutant phenotypes with *slo-1* mutants. These phenotypic similarities led to the identification of an interaction between the DAPC and BK channels in muscle, and the observation that BK channels are localized by the DAPC (Kim et al., 2009).

More recently, we have shown that the BK channel plays a role in the development of acute functional (within session) tolerance (AFT) to ethanol, but, importantly, that loss of *slo-1* does not eliminate the ability to develop AFT (Bettinger et al., 2012). This latter observation implicates other mechanisms of action for both ethanol's effects and for the development of tolerance to those effects. In this study, we showed that a triacylglycerol (TAG) lipase plays a significant role in regulating the rate of development of acute functional tolerance. By manipulating the levels of TAGs through a mutation in the TAG lipase, *lips-7*, we could alter the basal behavioral phenotypes associated with the hyperactivated gain-of-function mutations in the *slo-1* gene (Bettinger et al., 2012). This result provides genetic evidence for a link between lipid environment and BK channel function. Such an interaction between BK and the lipid environment has been well documented *in vitro* as having significant effects on the ethanol sensitivity of a BK channel (Crowley et al., 2003, 2005; Yuan et al., 2008, 2011a,b).

Dillon et al. (2013) made use of the *C. elegans* pharynx and associated neurons, a relatively closed neuromuscular circuit, to assess the effect of a concentration range of ethanol on the rhythmic pumping of the pharynx. In this system, *slo-1* is expressed only in the neurons. At low doses, with the *slo-1* null mutant, they did not see the typical stimulatory effects of ethanol, whereas at high concentrations, where ethanol inhibits pharyngeal activity, loss of *slo-1* had no effect. This may point to other targets of ethanol in the pharyngeal neurons or a direct action of ethanol on the pharyngeal muscle itself.

### *C. elegans*: use of genetics to identify an ethanol interacting domain in the SLO-1 BK channel

Recently, Davis et al. (2014) used a combination of forward and reverse genetics strategies to identify a domain on the BK channel that is ethanol responsive. This group took advantage of a large collection of 32 mutant strains carrying amino acid substitution mutations in the *C. elegans slo-1* gene, which they assayed *in vivo* for changes in ethanol sensitivity. The goal was to identify a mutation that altered ethanol sensitivity without altering BK channel function, with the idea that the mutation may specifically alter the ability of ethanol to directly interact with the BK channel. One mutation, resulting in a T381I substitution, produced significant resistance to the depressing effects of ethanol on the neuromuscular-controlled behaviors of egg laying and locomotion (Davis et al., 2014). The threonine affected by the mutation is conserved in mammals and flies and is located in the N-terminal portion of the RCK1 calcium-sensing domain. The substitution had very mild effects on normal SLO-1 functions; the only departure from wild-type phenotypes was a partial effect on sensitivity to an acetylcholinesterase inhibitor that is used as a measure of acetylcholine release. Therefore, the T381I mutation has a strong impact on ethanol sensitivity of the BK channel with little effect on normal channel function. The relevance of this amino acid residue across phyla was tested by creating an identical mutation in the human BK channel (T352I) and expressing that version in *C. elegans*. Transformation with the wild-type human BK channel could rescue *slo-1* null mutant animals, whereas the mutant human BK channel acted like the worm T381I mutant, it failed to restore ethanol sensitivity but did restore the other *slo-1* null mutant phenotypes (Davis et al., 2014). Single-channel patch clamp recordings of the human BK channel expressed in *C. elegans* neurons showed that in both the non-mutated and the mutated forms, currents could be detected that displayed all of the expected characteristics of the human channel, suggesting that the mutation did not alter normal BK channel properties. When these channels were expressed in HEK293 cells, only a minor difference in open probability across different voltages was detected between the non-mutated and mutant forms, suggesting the possibility of a subtle impact of the mutation on normal BK channel function, and furthermore, ethanol-induced potentiation was eliminated in the mutated human channel (Davis et al., 2014). The structural impact of the T381I mutation is not yet known but the equivalent amino acid in mammals (T352) is only 9 amino acids from the hydrogen-bonding lysine residue identified by Bukiya et al. (2014) as part of an ethanol interacting domain in the mammalian channel. The T381I mutation, which replaces a smaller polar residue with a larger nonpolar residue, may impact the same pocket either through hindrance of ethanol or through electrical charge dynamics.

### Drosophila: the role of the BK channel in rapid tolerance to ethanol

The fruit fly, *Drosophila melanogaster*, has proved to be an excellent model in which to carefully dissect the contribution of BK channels to the *in vivo* behavioral effects of ethanol. A single gene, *slowpoke*, encodes the BK channel, *slo*, in *Drosophila*, and its role in the response to alcohol sedation has been extensively characterized.

*slo* function in the fly response to drugs has been characterized primarily in an adaptive drug response phenotype, the development of rapid tolerance. These studies have used two drugs, ethanol and benzyl alcohol (BA). In these assays, flies are exposed to an ethanol or BA vapor in a closed chamber. Flies accumulate ethanol from the air and, after a brief initial startle activation response, become sedated (Rothenfluh and Heberlein, 2002), and similar kinetics are observed when flies are treated with BA (Ghezzi et al., 2004). When the flies are all sedated, the vapor is removed from the air flow, and the time that it takes the flies to recover the ability to stand is recorded. If the flies are then sedated a second time, they recover their postural control significantly more quickly than after the first sedation, a phenotype that is referred to as rapid functional tolerance (Ghezzi et al., 2004; Cowmeadow et al., 2005). Rapid tolerance is observable for at least 7 but fewer than 14 days after the initial sedation, and represents a long-lasting modification of nervous system function in response to the drug exposure. Sedation with benzyl alcohol or ethanol causes cross-tolerance with the other (Cowmeadow et al., 2006), strongly suggesting that the mechanism of rapid tolerance is the same for the two drugs.

*slo* function is required for the development of rapid tolerance, as *slo* null mutant animals do not become tolerant to either drug after an initial sedation (Ghezzi et al., 2004; Cowmeadow et al., 2005). This group has also been able to take advantage of an excellent genetic tool, the *ash*2^18^ deletion mutant, which removes only the promoter region driving expression of *slo* in neurons, but leaves the expression of *slo* in muscles intact, to map the requirement for *slo* function in rapid tolerance to the nervous system (Ghezzi et al., 2004; Cowmeadow et al., 2005).

Sedation with ethanol, BA, chloroform, CO_2_, cold temperatures, or sub-lethal doses of tetrodotoxin all cause a transient increase in *slo* mRNA levels in neurons that can be observed at 6 h post sedation (Ghezzi et al., 2004; Cowmeadow et al., 2006). This suggested that the drug-induced increase in *slo* expression may be responsible for the development of rapid tolerance, and indeed, when *slo* expression was induced with a heat-shock inducible transgene, the recovery kinetics from the first sedation resembled those of animals that had developed rapid tolerance (Cowmeadow et al., 2006).

Epigenetic modifications control this drug-induced increase in *slo* expression; these act on specific promoter sequences that are conserved across *Drosophila* species (Wang et al., 2007). There is a complex pattern of histone acetylation and deacetylation in the *slo* regulatory region that occurs over the course of 48 h after sedation (Wang et al., 2007), and these histone acetylation changes require binding of CREB to the *slo* promoter; loss of function of *Drosophila* dCREB2 eliminates drug-induced upregulation of *slo*, and eliminates rapid tolerance (Wang et al., 2009).

It is interesting that an increase in *slo* expression should cause a tolerance phenotype, which suggests that in this context, BK channels act to increase the excitability of cells. Ghezzi et al. (2010) explored the mechanism of this role of BK in tolerance to alcohol sedation in *Drosophila*. In these experiments, the firing capacity of the giant fiber neuron pathway was measured; the ability of the pathway to fire repeatedly is a measure of neuronal excitability (Tanouye and Wyman, 1980). An electrical stimulation was delivered into the eyes of the fly, and the firing of the giant fiber neuron pathway was measured in the dorsal-longitudinal flight muscles. Twenty-four hours after sedation with BA or ethanol, giant fiber neurons demonstrated an increase in excitability that was dependent on *slo* expression in neurons (Ghezzi et al., 2010, 2012). The interpretation of these results is that the drug-induced increase in *slo* expression causes a decrease in the refractory period in neurons, thereby increasing neuronal excitability. This increase in excitability acts to counter the depressing effects of BA and ethanol, and describes a potential mechanism for the development of rapid tolerance.

Additional evidence for this hypothesis comes from the observation that sedation with BA or ethanol also leads to an increase in the seizure susceptibility of the giant fiber pathway neurons that is due to the drug-induced increase in *slo* expression (Ghezzi et al., 2010, 2012), further indicating that these neurons have become hyper-excitable. This phenotype has characteristics in common with the seizure susceptibility that is sometimes observed in alcohol-dependent humans during alcohol withdrawal (Rogawski, 2005).

### Rodent expression studies: the effect of ethanol exposure on BK channel message levels

The two commonly used inbred mouse strains, C57BL/6J (C57) and DBA/2J (D2), show significant differences in their behavioral responses to ethanol and in their ethanol drinking preferences (Crawley et al., 1997). DBA/2J mice show significant ethanol-induced locomotor stimulation and reduced alcohol consumption relative to C57BL/6J. Kerns et al. (2005) took advantage of these behavioral differences, and examined global gene expression using microarrays in these two strains in the nucleus accumbens, prefrontal cortex, and ventral tegmental area 4 h following acute ethanol (2 g/kg) or saline injection. The BK channel encoding gene, *KCNMA1*, was one of only 307 genes found to be regulated in response to ethanol in either strain in any of the brain regions. *KCNMA1* expression was upregulated in the nucleus accumbens in D2 mice but was relatively unchanged in C57 mice. Wolen et al. (2012) further explored the differences between these two mouse strains, and increased the power of this approach by using the recombinant inbred BXD strains that were derived from an initial cross between C57 and D2 and then inbred for homozygosity to produce over 100 strains in which the original C57 and D2 genomes are recombined into unique combinations (Peirce et al., 2004). Wolen et al. (2012) used microarrays to examine global gene expression in the parent strains and in at least 27 of the BXD strains (depending on the brain region studied). They assessed gene expression in the nucleus accumbens, the prefrontal cortex, and the ventral midbrain 4 h following intraperitoneal injection of ethanol (1.8 g/kg) or saline, and found that *KCNMA1* gene expression was ethanol-responsive in all brain regions examined. In addition, expression of the *KCNMB4* gene, which encodes the β4 subunit of the BK channel in mammals, was found to be ethanol-responsive in the ventral midbrain. Focusing on the prefrontal cortex data, they identified networks of genes whose expression was highly correlated either basally, following ethanol exposure, or both. *KCNMA1* was identified as a hub gene based on the bioinformatic measures of connectivity and betweenness centrality in one of these large gene networks (Wolen et al., 2012). Hub genes are considered to be likely regulators of the transcriptional response to ethanol treatment so it is notable that *KCNMA1* falls into this category. The difference in *KCNMA1* regulation in response to ethanol in these different genetic backgrounds suggests that there is important genetic variation in the mechanisms of regulation in these strains, and points to important avenues for future study.

The increased expression of *KCNMA1* in ethanol-treated D2 mice is consistent with the *Drosophila* rapid tolerance studies, which showed increased BK channel expression following ethanol treatment (Cowmeadow et al., 2006; Ghezzi et al., 2010). In contrast, in a study of the effects of ethanol on *KCNMA1* gene expression, Pietrzykowski et al. (2008) showed that ethanol exposure of rat supraoptic nucleus (SON) neurons results in a down regulation of total *KCNMA1* mRNA. In primary cultured striatal neurons and in SON neurons in organotypic explants there is a rapid decrease in total *KCNMA1* mRNA levels within 15 min of ethanol (20 mM) exposure.

Pietrzykowski et al. (2008) also made the intriguing observation that ethanol exposure alters which splice forms of *KCNMA1* mRNA are present in these neurons. The authors examined the variety of alternatively spliced *KCNMA1* mRNAs and found that in untreated SON neurons, eight mRNA variants were detected, however, in 24 h-ethanol treated SON neurons, only two mRNA variants were found. These data suggest that ethanol is causing regulation of specific *KCNMA1* mRNAs. The rapid action (within 15 min of exposure) of ethanol on the level of *KCNMA1* message suggests that the regulation acts on preexisting mRNAs rather than on transcription levels. Particular targets of this down-regulation were mRNAs containing exon 29, which was dubbed ALCOREX (Pietrzykowski et al., 2008). Loss of mRNAs containing the ALCOREX exon is likely to impact the ethanol sensitivity of BK channels because when expressed transiently in HEK293 cells, BK channel isoforms that contain ALCOREX are more sensitive to the potentiating effects of ethanol and that potentiation is longer lasting than that in other isoforms lacking ALCOREX. To identify the mediators of the ethanol effect in down regulating specific *KCNMA1* mRNAs, the authors looked to regulation by specific microRNAs. Expression of miR-9 increases on a timescale equivalent to the down-regulation effects on *KCNMA1* mRNA. miR-9 is predicted to target ALCOREX-containing *KCNMA1* mRNAs based on the presence of and complementarity with an alternative 3'UTR (called 3'UTR-2.1) that is associated with ALCOREX-containing mRNAs (Pietrzykowski et al., 2008). These data identify miR-9 as a key regulator of BK channel ethanol tolerance although the *in vivo* consequences of this regulation remains to be tested, particularly in combination with beta subunit effects on sensitivity and tolerance.

### Mouse knockout studies: the role of the β subunits of the BK channel in the electrophysiological and behavioral responses to ethanol

The use of gene knockout techniques in mice allows for an *in vivo* examination of the function of a gene, including its role in behavioral responses. The knockout mutations used to examine the role of BK channel beta subunits have been particularly informative, although, as with any complete knockout in any organism, the consequences of loss of the gene must be interpreted with the possibility of compensation in the form of up or down regulation of other functionally interacting genes and/or the possibility that loss of the gene may result in developmental alterations.

There are four β subunits (β1–β4) that can associate with the BK alpha subunit to alter its physiological and pharmacological properties (reviewed by Torres et al., 2007; Pongs and Schwarz, 2010). These subunits show tissue specific expression, with some overlap. The subunits of particular importance to the effects of ethanol on BK channels are the β1 and β4 subunits, which are encoded in mammals by the *KCNMB1* and *KCNMB4* genes, respectively.

The β4 subunit is abundant in the CNS (Brenner et al., 2000; Weiger et al., 2000). Martin et al. (2008) examined the effect of β4 subunits on the development of tolerance to the effects of ethanol on BK channels. Expressed alone in HEK293 cells, the BK α subunit showed potentiation of activity by ethanol that diminishes within 10 min despite continuous ethanol exposure. In contrast, when the α subunit is co-expressed with the β4 subunit, tolerance to the potentiating effects of ethanol does not occur, suggesting that the β4 subunit is interfering with the mechanism of tolerance without altering the potentiating effect of ethanol. This outcome was replicated with medium spiny neurons from the mouse ventral striatum in wild-type and *KCMNB4* (β4) knockout animals, in which activation of the BK channel depresses excitability of the neurons, decreasing the number of action potentials (APs) evoked by current injection in striatal slice preparations. Loss of β4 allowed tolerance to develop to potentiating effects of ethanol on the BK channel (Martin et al., 2008). In slices from wild-type mice, ethanol decreased the number of APs for at least 8 min of recording, whereas in slices prepared from β4 knockout mice, the degree of ethanol-induced reduction in APs diminished over the course of the 8 min of recording, showing that tolerance could develop to the effect of ethanol in the absence of β4 subunits (Martin et al., 2008). Two behavioral assays were then performed using the β4 knockout mice. First, the sensitivity to ethanol intoxication was measured. C57BL/6 mice show significantly decreased rates of acute (5–15 min) locomotor activity in the presence of 2 mg/kg ethanol. In wild-type mice, tolerance to this effect is observable at the 15 min post-injection time point, but only after 4 days of identical treatment (Martin et al., 2008). This effect somewhat resembles the rapid tolerance displayed by *Drosophila* that have seen a previous ethanol exposure (Cowmeadow et al., 2006). In contrast, β4 knockout mice showed significant within-session acute functional tolerance (AFT) to the decreased speed of locomotion effects of ethanol at the 10 and 15 min time points, even on the first day of ethanol administration (Martin et al., 2008), an effect that resembles the development of AFT seen in *C. elegans* for the effect of ethanol on locomotion (Davies et al., 2004; Bettinger et al., 2012). The simplest explanation for these outcomes is that the β4 subunit has a negative effect, *in vivo*, on the development of tolerance to the potentiating effects of ethanol on the BK channel, and that tolerance can only develop slowly in the presence of β4.

To examine the impact of the lack of tolerance associated with β4 knockout on drinking behavior, Martin et al. (2008) went on to assess voluntary ethanol drinking using a “drinking in the dark” paradigm, which restricts access to ethanol. The β4 knockout mice consumed greater quantities of ethanol during the first three access periods and achieved a higher blood alcohol level than the wild-type mice, without changing their water intake. This increased level of ethanol drinking might reflect a decreased sensitivity to one or more effects of ethanol as tolerance to the drug would be predicted to be greater in the β4 knockout animals.

A separate genetic study has confirmed that the presence of particular β subunits can alter the direction of ethanol's effect on BK channels, such that inhibition of the BK channel by ethanol is also possible. The β1 subunit is highly expressed in smooth muscle (Behrens et al., 2000). Focusing on ethanol's effects on smooth muscle, Bukiya et al. (2009) examined the requirement for the β1 subunit for ethanol-induced inhibition of BK channels in myocytes that results in cerebrovascular constriction. Depending on the intracellular Ca^2+^ concentration, ethanol can potentiate (lower Ca^2+^) or inhibit (higher Ca^2+^) BK potassium currents. When associated with a β1 subunit, the BK channel is inhibited at significantly lower intracellular calcium concentrations (Bukiya et al., 2009). A comparison of the effects of ethanol on cerebral artery myocytes derived from wild-type and *KCNMB1* (β1) knockout mice showed that the β1 subunit was able to completely change the effect of ethanol on the BK channel. In wild-type myocytes, ethanol inhibited BK channel activity, whereas in the absence of the β1 subunit, ethanol potentiated BK channel activity. An effect on the contraction of cerebral arteries that is consistent with the β1 subunit promoting BK channel inhibition was also observed; arteries from β1 knockout mice fail to contract with ethanol exposure while arteries from wild-type animals (β1-containing) showed a 10-15% reduction in diameter (Bukiya et al., 2009).

While the β1 subunit is highly expressed in smooth muscle (Behrens et al., 2000) the β1 subunit also has limited expression elsewhere, including the brain (Martin et al., 2004). Kreifeldt et al. (2013) examined the impact of β1 or β4 knockouts in a C57BL/6J genetic background on voluntary ethanol consumption in mice. In a paradigm of voluntary ethanol consumption, two-bottle choice continuous access followed by intermittent access, loss of β1 and β4 had no effect on drinking levels. Note that this is seemingly in contrast to the data presented above, where β4 knockout mice consumed more alcohol during a restricted access (drinking in the dark) paradigm (Martin et al., 2008). It is possible that the drinking paradigms used in the two studies were sufficiently different to expose a mutant phenotype in one paradigm but not the other. An alternative possibility that is proposed, is that genetic background of the control strains used in the Martin et al. (2008) study may differ from the β4 knockout animals that were tested (Kreifeldt et al., 2013). The most interesting outcome of the comparison of β1 and β4 knockout mice was found when the mice were first made ethanol-dependent before their ethanol consumption was assessed. Chronic intermittent exposure (CIE) to ethanol vapor with repeated withdrawals produces symptoms of physiological ethanol dependence, and when these treated mice are then given limited access to ethanol they have been shown to escalate their ethanol consumption during the limited access periods over time (Becker and Lopez, 2004). When β1 and β4 knockout mice were made ethanol dependent by CIE treatment, the β1 knockout mice were found to escalate their ethanol consumption to a greater extent than the wild-type mice, while the β4 knockout mice had a lower rate of escalation of consumption than the wild-type animals (Kreifeldt et al., 2013). This surprising combination of phenotypes highlights both the complexity of the ethanol drinking phenotypes and the complex roles of BK channels in those behaviors. Simplistically, one might have predicted that loss of β1 should increase ethanol sensitivity because the presence of β1 prevents potentiation of BK by ethanol. In contrast, loss of β4 should decrease ethanol sensitivity via the rapid development of tolerance; β4 prevents the development of tolerance and, in its absence, tolerance to ethanol's effects can occur more readily. As appears to be the case in human populations (Schuckit and Smith, 1996), an increase in sensitivity to ethanol may be predicted to decrease voluntary drinking, whereas a decrease in sensitivity may increase drinking behavior. However, this model is clearly too simple. The combination of the adaptive changes that must be occurring with the generation of ethanol dependence and the as yet not fully understood roles for the beta subunits in modifying BK channel function, as well as the potential for different tissue specific subunit localization, makes a complete interpretation of this data difficult. It is clear, however, that beta subunit availability is an important factor in specific ethanol effects and in drinking behaviors.

### Human population studies: association of BK channel genes with alcohol response phenotypes and alcohol dependence

Ultimately, the goal of the work in the model organisms is to develop an understanding of the relevant mediators of the ethanol response that can be applied to the human alcohol response. Alcohol dependence (AD) is a complex phenotype, and its development is influenced by many factors. Genetics plays a significant role in the risk for AD (Prescott and Kendler, 1999) and much effort has been focused on identifying “liability loci,” alleles that predispose individuals to developing AD. Despite several large-scale genome wide analyses for genes associated with AD, few good candidates have been identified, and almost none, with the exception of metabolic enzymes, have been replicated in more than one study. This is likely to be due, at least in part, to the complex genetic architecture of risk for AD (Kendler et al., 2012), which suggests that there will not be individual genes that have a very large effect on liability, but rather that there will be many genes that have small, but real, effects. A second complexity in these analyses is that for phenotypes such as an increased liability to develop alcohol dependence, the severity of effects of the alleles on gene function are likely to be somewhat subtle. That is, in the model organism studies, we are able to strongly decrease or strongly potentiate function of a gene, whereas in human populations, allelic variation is likely to cause more minor changes in gene function; the allelic variation in human populations will include change of function mutations, and completely null mutations will be more rare. Taken together, this suggests that any signals that are detected are likely to be weak. Therefore, weaker signals, in particular those that appear in more than one study, may identify biologically relevant genes for AD. It is particularly intriguing, therefore, that signals have been reported in the BK channel-encoding gene, *KCNMA1*, in several different human population studies.

In 2005, Schuckit et al. reported a study of a population of sibling pairs in which they used linkage analysis to identify association of particular chromosomal regions with alcohol response phenotypes. They studied the level of response to alcohol, which is the degree of effect of a given dose of alcohol on objective and subjective measurements. The most consistent results were found for the Subjective High Assessment Scale (SHAS) measure with linkage on chromosome 10. The investigators looked specifically SNPs in the *KCNMA1* gene locus, which is within the chromosome 10 interval of interest. Six of 44 SNPs produced statistically significant association with the SHAS measure, but were not significant after correction for multiple testing, possibly due to a small sample size.

A second signal in *KCNMA1* was detected by Kendler et al. (2011) in a genome wide association study (GWAS) for alcohol dependence symptoms. In this study, the most significant intragenic single nucleotide polymorphism (SNP) in a European-American population was in the *KCNMA1* gene, which was accompanied by multiple additional SNPs with low *p* values. The finding of several nominally associated SNPs within the same gene lends support to the observation of association with *KCNMA1*. However, as with the Schuckit et al. (2005) study, none of the SNPs identified in this study (for any gene) approached genome-wide significance.

Edenberg et al. (2010) found in a GWAS of alcohol dependence using the COGA (Collaborative Study on the Genetics of Alcoholism) that among the top 985 SNPs that were highly ranked for association with alcohol dependence in European Americans were six SNPs that showed nominal (*p* < 0.05) significance in a smaller African American case-control study. One of these SNPs was in the *KCNMA1* gene. In this study, again, no SNP in any gene met the criteria for genome-wide significance. Edenberg et al. (2010) also found a SNP in *KCNMA1* was nominally significant (*p* < 0.05) for early-onset alcohol dependence in a family-based association analysis.

Recently, Han et al. (2013) used an approach that combined GWAS data sets (COGA and the Study of Addiction: Genetics and Environment (SAGE)) with human protein interaction networks to identify modules of networked genes that were enriched for highly ranked genes associated with alcohol dependence. When they tested their final 39-gene network, which included both *KCNMA1* and *KCNMB1*, for association with alcohol dependence, they found significant associations in both European Americans and African Americans in the merged COGA and SAGE GWAS data sets. The association of the gene network with AD was replicated in two European American samples and one African American sample.

## Conclusions

Genetic studies in *C. elegans, Drosophila melanogaster*, and mice have all demonstrated that BK channels are central to the behavioral effects of ethanol across these diverse phyla. It is interesting to note that while an important role for BK in ethanol response behaviors is apparently conserved, the actual behavioral outcomes of altering the function of BK channels are different across these models, which points to the complexity of the effects of ethanol on BK channels, and to the complexity of the roles of BK channels in ethanol response behaviors.

In *C. elegans* and in *Drosophila*, the roles for BK channels in ethanol response behaviors appear to be both strong and reasonably straightforward, although these effects are quite different in the two models. In worms, the activation of the *slo-1* BK channel by ethanol is a primary mechanism of the initial sensitivity to the intoxicating effects of ethanol, and loss of *slo-1* causes resistance to ethanol. Worm BK channels are also likely to be negatively modulated to develop acute functional tolerance, and this modulation may involve modification of the lipid bilayer. In sharp contrast, in flies, ethanol induces expression of BK, and this increase in BK activity leads to a decrease in sensitivity to ethanol, this can be observed as resistance to ethanol when *slo* is induced experimentally, or as rapid tolerance when ethanol induces the response.

In mammals, the story is substantially more complex than it is in either of the invertebrate models. BK channels can be activated or inactivated by ethanol, depending on calcium levels and the presence or absence of specific β subunits, and there is dynamic regulation of the channels during the development of tolerance. The differential expression of *KCNMA1*, either basally or in response to ethanol treatment, in two inbred mouse strains and their progeny that display different behavioral responses to ethanol, points to the importance of genetic background and genetic factors that could regulate levels and localization of the BK channel and its β subunits. Very acutely, *KCNMA1* expression in rats is decreased by miR-9 in response to ethanol treatment, but induction of *KCNMA1* has been observed in mice in separate studies in specific brain regions 4 h after an injection of ethanol. Loss of different β subunits can alter drinking behavior in opposite directions. All of these observations highlight the complex relationship between the alpha and beta subunits, the tissues where they are expressed, and the dynamic changes that can occur with ethanol treatment.

Together, these observations across different organisms and experimental paradigms all highlight the importance of BK channels in the behavioral response to ethanol. It may be that the invertebrate models may each feature a different aspect of the rich mammalian involvement of BK in ethanol behavioral responses.

One goal of studies of the molecular effects of ethanol on neuronal function is to ultimately be able to identify and intervene with individuals who are at high genetic risk of developing an alcohol use disorder before they begin to abuse the drug. BK channels, therefore, represent an excellent candidate for examination in studies of genes that impact the genetic liability to develop an abuse disorder. Several human studies have found suggestive evidence for allelic variation in BK channels being associated with alcohol dependence. Each of these signals individually failed to achieve genome wide significance, but this is perhaps not surprising, given the difficulties that have been encountered in identifying AD liability loci across studies. Indeed, the repeated finding of nominally significant signals in *KNCMA1* in many different studies lends support to each individual result. Together, these data strongly support a model in which allelic variation in the human BK channel-encoding gene is a risk factor for developing AD.

The genetic and molecular analyses of BK channels are a rich resource for suggesting additional candidate genes for association with AD in human population studies. If variation in BK channels themselves can modify liability, as is suggested by the repeated identification of *KCNMA1* in human studies, then it seems likely that variation in modifiers of BK function would also be potential liability loci. The well-established roles for the microRNA miR-9 and the BK β subunits in altering BK function, and, specifically, in modifying the ethanol responsiveness of BK, make them excellent targets for study. In addition, we are particularly intrigued by the genes that regulate the lipid milieu in which the BK channels reside.

An additional goal of these studies is to provide information for the rational development of pharmaceutical interventions for alcohol dependence. The recent identification of specific ethanol interaction domains in the BK channel in both worms and mammals (Bukiya et al., 2014; Davis et al., 2014), provides promising specific targets for the development of molecules that prevent the BK mediated effects of ethanol.

Finally, there remains much that we do not know about the roles of BK channels in responses to ethanol. Very recently, there has been an exciting report describing a newly identified function of BK channels in the nucleus of neuronal cells. Li et al. (2014) report that functional BK channels reside on the nuclear envelope of mammalian hippocampal neurons. Their finding that blockade of BK function can affect nuclear Ca^2+^ levels and modify activity dependent gene transcription opens an entirely new field of study of the role of BK channels in the effects of ethanol exposure on brain function.

### Conflict of interest statement

The authors declare that the research was conducted in the absence of any commercial or financial relationships that could be construed as a potential conflict of interest.
